# Hydrogels as a Potential Biomaterial for Multimodal Therapeutic Applications

**DOI:** 10.1021/acs.molpharmaceut.4c00595

**Published:** 2024-09-18

**Authors:** Harpreet Kaur, Bishmita Gogoi, Ira Sharma, Deepak Kumar Das, Mohd Ashif Azad, Devlina Das Pramanik, Arindam Pramanik

**Affiliations:** † Amity Institute of Biotechnology, Amity University, Noida 201301, India; § Department of Chemistry and Nanoscience, GLA University, Mathura, Uttar Pradesh 281 406, India; ∥ School of Medicine, University of Leeds, Leeds LS97TF, United Kingdom

**Keywords:** Cancer immunotherapy, Injectable hydrogels, Periodontitis, Smart hydrogels, Tissue regeneration, Wound healing

## Abstract

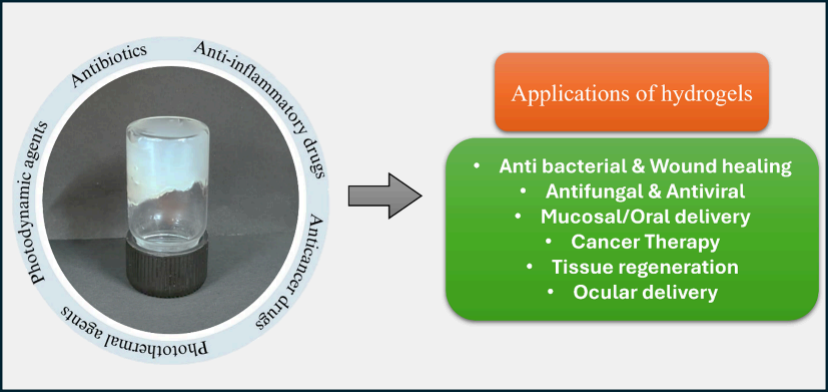

Hydrogels, composed of hydrophilic polymer networks, have emerged as versatile materials in biomedical applications due to their high water content, biocompatibility, and tunable properties. They mimic natural tissue environments, enhancing cell viability and function. Hydrogels’ tunable physical properties allow for tailored antibacterial biomaterial, wound dressings, cancer treatment, and tissue engineering scaffolds. Their ability to respond to physiological stimuli enables the controlled release of therapeutics, while their porous structure supports nutrient diffusion and waste removal, fostering tissue regeneration and repair. In wound healing, hydrogels provide a moist environment, promote cell migration, and deliver bioactive agents and antibiotics, enhancing the healing process. For cancer therapy, they offer localized drug delivery systems that target tumors, minimizing systemic toxicity and improving therapeutic efficacy. Ocular therapy benefits from hydrogels’ capacity to form contact lenses and drug delivery systems that maintain prolonged contact with the eye surface, improving treatment outcomes for various eye diseases. In mucosal delivery, hydrogels facilitate the administration of therapeutics across mucosal barriers, ensuring sustained release and the improved bioavailability of drugs. Tissue regeneration sees hydrogels as scaffolds that mimic the extracellular matrix, supporting cell growth and differentiation for repairing damaged tissues. Similarly, in bone regeneration, hydrogels loaded with growth factors and stem cells promote osteogenesis and accelerate bone healing. This article highlights some of the recent advances in the use of hydrogels for various biomedical applications, driven by their ability to be engineered for specific therapeutic needs and their interactive properties with biological tissues.

## Introduction

Hydrogels are three-dimensional porous networks that mimic the extracellular matrix of living tissues by absorbing huge volumes of water or biological fluids.^1^ In 1894, the term “hydrogel” was first used to describe a colloidal gel made of inorganic salts.^2^ The actual hydrogel concept emerged later in 1960 with the introduction of polyhydroxyethyl methacrylate, a material known for its strong water affinity and intended for continuous contact with human tissues. The development of patient-centric materials by hydrogels resulted in a notable upsurge in biomedical research, particularly during the 1970s.^2^ Hydrogel development has progressed through three generations. Initially, materials with high swelling and good mechanical properties were created by focusing on the chemical changes. A second generation of hydrogels came up later with the ability to react to various stimuli such as temperature, pH, or concentration of molecules.^3−5^ These particular stimuli can cause the material to exhibit certain properties, like polymerization, the administration of a drug, or the development of an in situ pore.^6^ Poly(ethylene glycol)-poly(lactic acid) interaction and other physical cross-linking techniques are among the most complicated materials that were explored in the third generation.^7,8^ The development of so-called “smart hydrogels”, polymeric matrixes with a broad range of adjustable characteristics and trigger stimuli, has recently attracted attention for the application of hydrogels in the biomedical field.^9,10^ They are perfect for use in engineering and medicine due to their hydrophilic nature and biocompatibility.^11,12^

Hydrogels can be classified into 3 types based on the source of material used, i.e., natural, synthetic, and hybrid. Poly(lactic acid) (PLA), poly(vinylpyrrolidone) (PVP), polycaprolactone, poly(vinyl alcohol) (PVA), and poly(ethylene glycol) (PEG) are some of the polymers used for synthesis of synthetic hydrogels.^13^ Some of the natural polymers used for hydrogel production are cellulose, chitosan, chitin, starch, alginate, carrageenan, etc.^13^ Physical and chemical cross-linking procedures are the two main types of hydrogel formation processes. Physical cross-linking is the process of creating noncovalent bonds, such as hydrogen bonds, hydrophobic bonds, and ion interaction, which results in products that can be reversibly produced or disrupted.^14−16^ Contrarily, hydrogels with chemical cross-linking are composed of polymers joined by covalent bonds or certain cross-linking agents that can be activated by light and intense radiation. Compared to physical hydrogels, these chemical cross-linked hydrogels are more heat robust and generate stronger bonds.^16^ Till now, numerous hydrogels made of natural polymers with unique functional interfaces have been developed.^17,18^ These biomaterials’ superior mechanical qualities, such as their flexibility, swelling capacity, and speedy transport of waste and nutrients, make them appealing candidates for regenerative medicine.^19^ A desirable hydrogel must restore tissues and offer the best conditions for cell proliferation, vascularization, and host integration in order to aid in wound healing.^20^ Because of their very porous structure, hydrogels are used in drug delivery systems to provide a sustained release of medication.^21^ Hydrogels are especially useful as a drug delivery system because they keeps drugs from degrading and eliminate their negative effects, such as short half-lives and poor water solubility. Benefits from the usage of hydrogels in drug delivery include fewer adverse effects, effective drug utilization, precise drug targeting to certain areas, and inexpensive prices.^22^ Hydrogel films have a greater surface area than hydrogel particles and can be used to bandage sores to protect them while they are being treated.^23^ Additionally, nanogels that are water-based dispersions at the nanoscale created by cross-linking polymers could be used as drug delivery carriers for in vivo administration. Nanogels can enhance the bioavailability of insoluble pharmaceuticals, accomplish regulated and prolonged discharge drug administration, allow focused drug administration, and act as transporters for bioactive macromolecule medications.^24,25^ To address various treatment aspects, injectable hydrogels are found to be suitable for both local and multitime-scale administration locations.^7,26,27^

Hydrogels aid in antimicrobial wound healing and tissue regeneration.^28−33^ They create a moist environment, which is crucial for healing, and can be embedded with antimicrobial agents and bioactive compounds, helping to reduce infection risks.^10,34^ Additionally, hydrogels can be tailored to release therapeutic agents in a controlled manner, further promoting wound healing and reducing inflammation. Hydrogels also support cellular activities necessary for tissue repair such as cell migration and proliferation. Their tunable mechanical properties allow for customization to match the target tissue’s stiffness, enhancing integration and function. Additionally, their porous structure allows for efficient oxygen and nutrient exchange, enhancing tissue regeneration such as in cardiac tissue, bone, spinal cord, etc.^32,33,35−37^ In cancer immunotherapy, hydrogels play a crucial role in delivering therapeutic agents such as immune checkpoint inhibitors or cytokines directly to the tumor site.^38^ By encapsulation of these agents within hydrogel matrices, their release can be controlled, leading to sustained and localized delivery. This targeted approach minimizes systemic side effects while maximizing therapeutic efficacy, ultimately improving patient outcomes.^39^ In ocular drug delivery, hydrogels offer a noninvasive and patient-friendly method for administering drugs to treat various eye conditions.^40^ Hydrogel-based ocular inserts or contact lenses can release drugs gradually, ensuring prolonged therapeutic levels in the eye while reducing the need for frequent administration.^41^ Furthermore, the mucoadhesive properties of hydrogels enable prolonged retention on the ocular surface, enhancing the drug bioavailability and efficacy. Similarly, in oral drug delivery, hydrogels address challenges such as poor solubility, enzymatic degradation, and low bioavailability of drugs.^42^ Hydrogel-based oral formulations can protect drugs from degradation in the harsh gastrointestinal environment, promote sustained release, and enhance absorption through mucosal membranes.^43^ Moreover, they can be engineered to respond to specific stimuli, such as pH or enzymes, enabling controlled drug release at the desired site within the gastrointestinal tract.^4^ This review will highlight some of the recent interesting applications of bioactive hydrogels in the field of antibacterial wound healing, oral delivery of drugs, cancer immunotherapy, tissue regeneration, and similar potential biomedical aspects.

## 2 Antibacterial Hydrogels for Wound Healing

Certain bacteria such as *Escherichia coli*, *Acinetobacter baumannii*, *Staphylococcus epidermis*, *Pseudomonas aeruginosa*, and *Staphylococcus aureus* inhabit chronic wounds that could have severe effects on the patient.^44^ In many kinds of wounds, such as burns and traumas, opportunistic bacteria can invade, colonize, and multiply at the wound site, which may result in infection.^39,45^ Infected wounds can lead to a protracted wound healing process as well as, in certain situations, disability and death.^24^ Some multidrug-resistant (MDR) bacteria have surfaced recently because of antibiotic overuse.^46,47^ For instance, *P. aeruginosa* isolates have been shown to have enhanced resistance to several antibiotic classes, including carbapenems, aminoglycosides, and quinolones.^48^ Thus, the use of antibiotics as a treatment is becoming ineffective in preventing infections.^49^ Developing novel approaches and technologies that make use of antibiotics or antibacterial compounds is crucial for preventing antibiotic resistance and lowering mortality.

In this regard, hydrogels are ideal candidates for wound dressing due to their multifunctional qualities.^50−52^ These multifunctional qualities include being biodegradable, biocompatible, and made of inflated 3D porous networks, and they are potential wound dressing materials due to their efficient capacity for water retention, microbial barrier properties, hydration, and wound healing acceleration.^25,53,54^ These hydrogel materials are nontoxic and nonallergic and offer good oxygen transmission between the wound site and the atmosphere. Therefore, the healing of body tissues should be possible with a suitable hydrogel which would induce cell growth, proliferation, and vascularization.^10^ Scaffold deterioration, however, ought to occur during or just after the healing process. Additionally, the right hydrogels with a high porosity rate and a good swelling ability may be employed as a delivery vehicle to regulate the release of antibacterial drugs.^55^ As an example, Garcia et al. developed casein-based hydrogels of sodium salt loaded with antibiotic drug “polyhexanide” which showed efficient biocompatibility, controlled release of drug for 48 h, and potential antibacterial activity against *P. aeruginosa* and *S. aureus*.^56^ Similarly, keratin-peptide-based hydrogel has demonstrated drug-resistance reversal when administered with antibiotic on multidrug-resistant bacteria by downregulating drug efflux pump.^57^ Further this antibiotic–hydrogel combination showed effective wound healing. Altunbas et al. encapsulated curcumin into injectable peptide hydrogel.^58^ This curcumin-encapsuled peptide hydrogel induced apoptosis in medulloblastoma.^58^ For 3D neural tissue culturing, Semino et al. performed a ground-breaking work on entrapping neural cells in peptide fibrous hydrogels.^59^ Similarly, peptide amphiphile hydrogels have demonstrated the ability to regulate stem cell development and treat cancer.^60,61^ Thus, many different types of biomaterials (synthetic or natural) have been utilized for developing hydrogels for biomedical applications.

**Figure 1:**
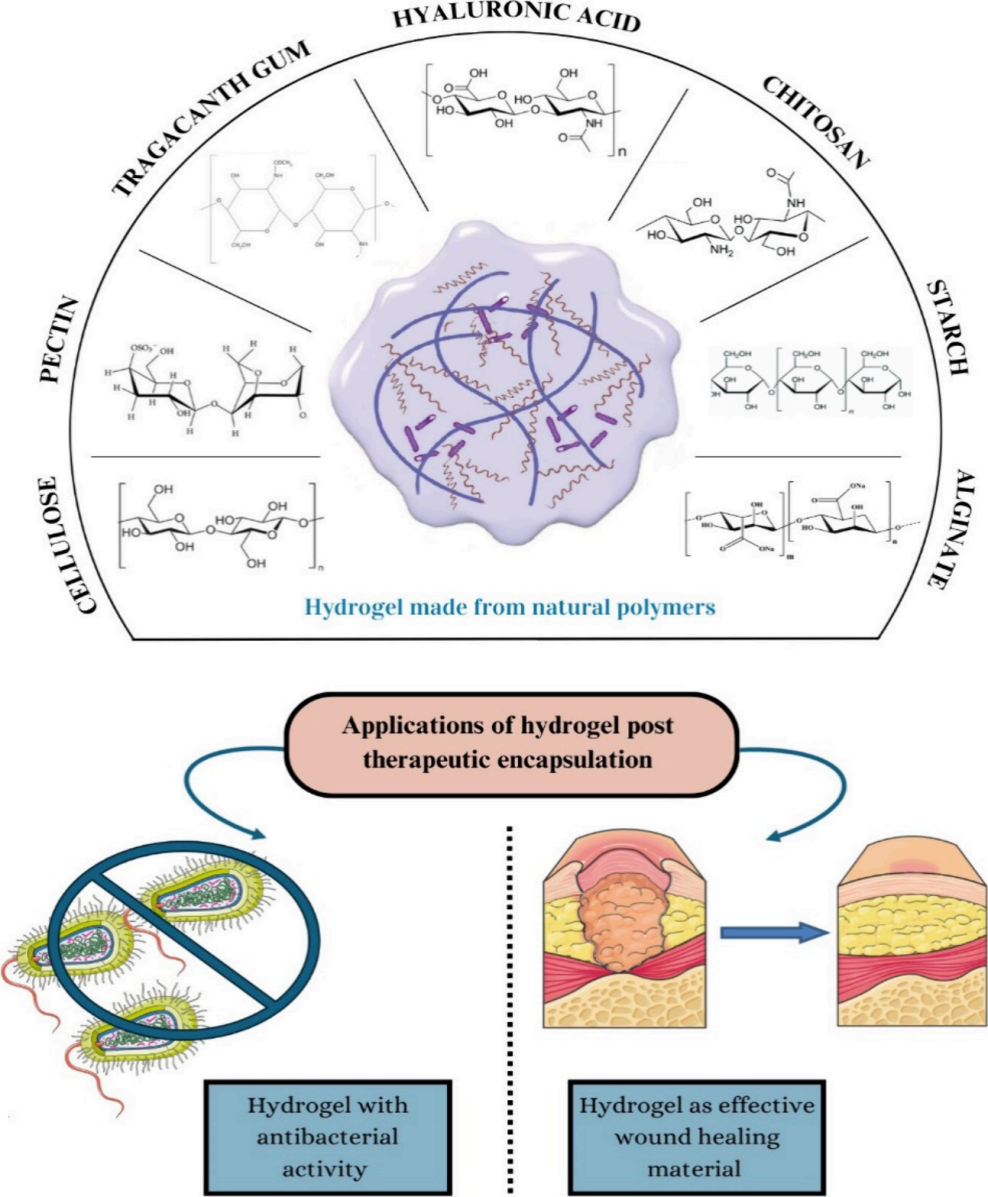
Types of natural polymers used from hydrogel preparation, and its application as an antibacterial agent for wound healing. Parts of the figure were drawn by using pictures from Servier Medical Art. Servier Medical Art is licensed under CC BY 4.0.

However, much attention has been gained recently on hydrogels based on polymers (alginate, dextran, chitosan, pectin, chitin, cellulose) that are found naturally in the extracellular matrix (ECM) due to their biocompatibility, nontoxicity, and being cost effective.^62,63^ The next section will highlight some of these natural polymer-based hydrogels as antibacterial agents for wound healing applications (Figure 1).

### 2.1 Chitosan-Based Hydrogels

**Figure 2:**
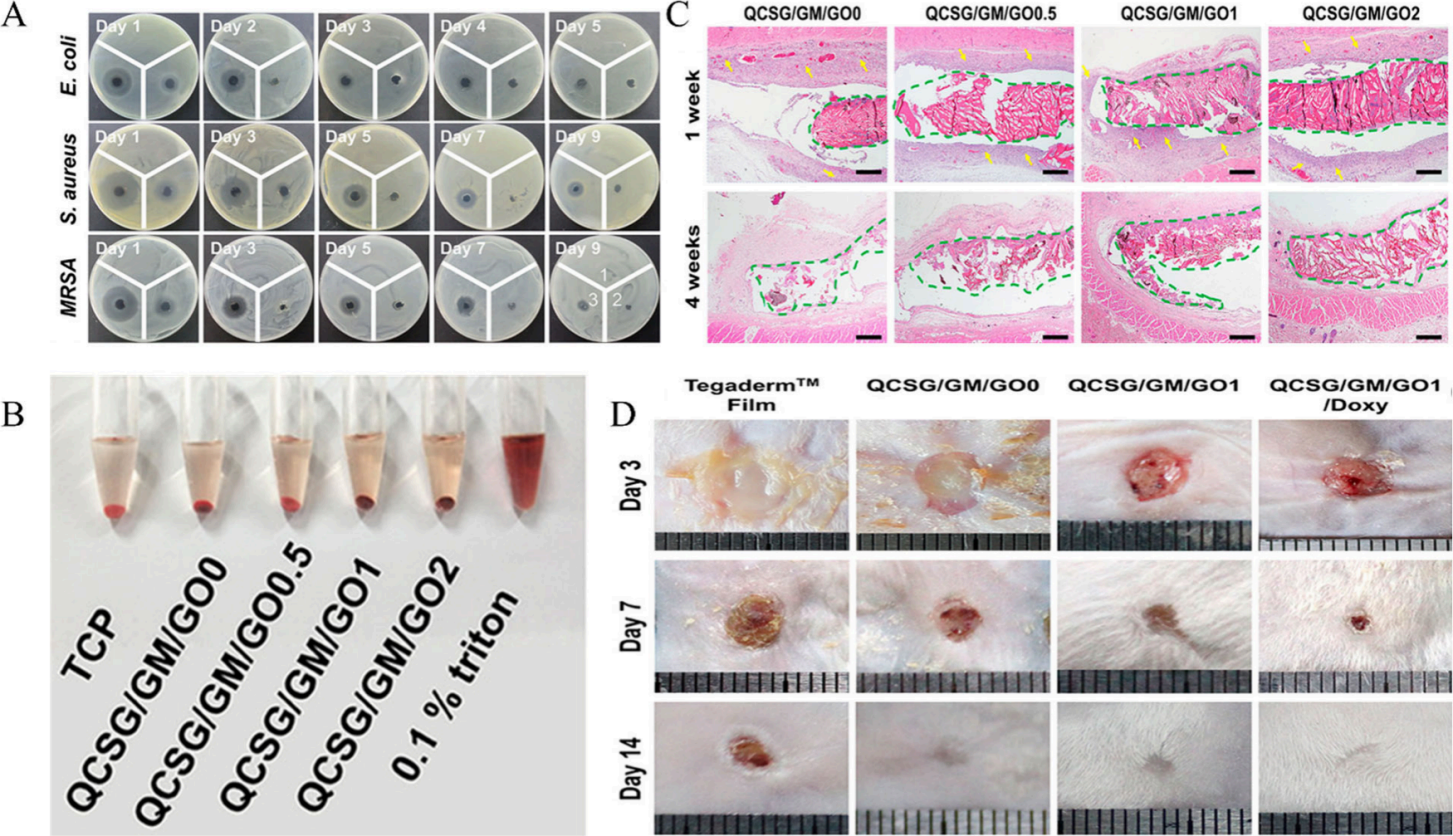
(A) Inhibition zones on three strains of bacteria, 1, 2, and 3, representing blank, chitosan hydrogel, and chitosan hydrogel loaded with doxycycline, respectively, showing the effective antibacterial activity of chitosan hydrogel loaded with doxycycline. (B) Hemolytic activity assay of the hydrogels where 0.1% triton is a positive control. (C) Histocompatibility with HE staining of skin tissue after hydrogel incubation at different weight ratios. (D) In vivo wound healing outcome on the 3rd, 7th, and 14th days for different groups showing doxycycline loading having effective would closure. Adapted with permission from ref (67). Copyright 2020 American Chemical Society.

Chitosan, a natural cationic polymer, is composed of two monomer units: *N*-acetyl-d-glucosamine (an aceylated unit) and β-(1 → 4)-linked d-glucosamine (a deacylated unit). It has strong film forming capability and has great qualities such as low toxicity, biodegradability, and biocompatibility.^64^ Chitosan has a strong antibacterial effect because of its cationic composition, which makes it easier for it to bind to negatively charged bacterial membranes and cause intracellular proteins and other constituents to leak out. Chitosan inhibits fibrin breakdown during hemostasis while promoting platelet and erythrocyte aggregation.^65^ Second, it can aid in the removal of bacteria from the inflammatory wound site. Lastly, it can encourage the development of granulation tissue, which speeds up skin proliferation. Chitosan is therefore often used as a healing substance for wound dressings.^66^ Because of its advantageous qualities, chitosan is said to be the most useful and necessary ingredient for creating hydrogels. Because its amino and hydroxyl groups may be easily chemically modified and conjugated with other biomolecules, it permits an improved biological performance for wound healing activity. However, chitosan’s limited mechanical strength and poor solubility make it unsuitable for use in wound dressings on its own. This limitation could be overcome by quaternization and grafting. Liang et al. synthesized antimicrobial conductive hydrogels for infection wound healing by functionalizing quaternized chitosan with encapsulated graphene oxide and glycidyl methacrylate and loading with doxycycline.^67^ These hydrogels show an outstanding ability to repair wounds and prevent infectious skin tissue abnormalities. During the in vivo trail, the hydrogel showed improved angiogenesis, suppressed bacterial growth and colony formation, and decreased scar formation (Figure 2).

Wei et al. created a range of adhesive hydrogels using functional groups grafted on carboxymethyl chitosan (CMCS).^68^ Because of the phenol and quaternary ammonium groups grafted on the CMCS, these hydrogels exhibit strong antioxidant and antibacterial characteristics. Their superior hemostatic properties, strong adsorption ability, and cytocompatibility were demonstrated in vitro, indicating their high potential. Multifunctional hydrogels have demonstrated a significant capacity for vascularization and collagen deposition during in vivo assessment findings, indicating their potential for application as a wound healing material. Thermosensitive hydrogels have been developed using catechol functionalization. These hydrogels led to faster wound healing by encouraging cell migration, producing new collagen fibers and blood arteries, and preventing cell accumulation.^69^ These studies indicate the potential of the chitosan-based hydrogels as a promising wound healing dressing.

### 2.2 Alginate-Based Hydrogel

Alginate is an anionic polysaccharide present in brown algae.^70^ Because alginate has a strong absorptivity, it has an affinity toward pathogenic bacteria and therefore helps in inhibiting infections. Additionally, it promotes tissue granulation and cell proliferation,^71^ which accelerates the healing of wounds. Alginate-based materials could be useful in the treatment of both acute and chronic wounds.^72,73^

An alginate-based hydrogel membrane with thermosensitive properties was developed for effective and faster wound healing. The membrane showed better tensile strength and mechanical properties and excellent swelling properties. It significantly inhibited the bacterial growth of *S. aureus* and *P. argenosa* species. A similar hydrogel formulation based on alginate was developed by Zhang et al.^29^ The hydrogels showed 150% swelling and 80% porosity and were biologically compatible with various mammalian cell lines. This hydrogel showed excellent antibacterial activity against a broad range of bacterial strains, including pathogenic ones. In the rat scald model, the hydrogel formulation revealed improved wound healing action, preventing infection, pointing to the potential use of hydrogels in wound care.

Zhang et al. synthesized injectable hydrogels using a combination of chitosan (CA) and alginate (AG).^51^ The hydrogels disintegrated and released the drug faster but also broke down more quickly when administered subcutaneously, which led to increased tissue buildup. By promoting angiogenesis and enhancing blood vessel development, the hydrogels also accelerated wound healing.^51^ Oh et al. created a different hydrogel of CA-AG with gelatin, showing significant wound healing activity without any toxicity.^74^ These studies indicate that a polysaccharide-based hydrogel approach may be vital to wound care and highlight the potential of alginate hydrogels for effective wound healing.

### 2.3 Hyaluronic Acid-Based Hydrogels

Hyaluronic acid (HA) is a hydrophilic, nonsulfated, anionic biocompatible polymer found in various biological sources such as cartilage, skin, vitreous humor, and cockscomb.^75^ HA has weak mechanical performance and degrades quickly through oxidizing agents and enzymatic degradation. In order to overcome these limitations, HA can be grafted, loaded with growth factors, chemically altered, or made into functionalized hydrogels for faster wound healing.

Wang et al. developed a HA-based hydrogel containing dopamine hydrochloride which demonstrated effective mechanical and wet adhesion strength.^30^ In vivo study on rat models showed the hydrogel’s cellular biocompatibility produced enhanced homeostatic effects. Rapid skin healing of wounds was facilitated by the hydrogel through the synthesis of collagen and granulation tissue. As an outcome of this study, it was found that due to scallops’ strong adherence, this hydrogel may be used in place of sutures for wound therapy.^30^ To create photosensitive hydrogels, Zhao et al. combined hyaluronic chain encapsulation of epidermal growth factor (EGF) with supramolecular polysaccharide acid.^28^ The hydrogel demonstrated enhanced angiogenesis and faster growth of granulation tissue by delivering EGF to the wound site. Similarly, Yang et al. developed hydrogels based on HA which improved wound healing by encouraging collagen deposition and angiogenesis in the injured area.^76^ These ECM-encapsulated hydrogels could have a significant positive impact on the medical field.

### 2.4 Cellulose-Based Hydrogels

Cellulose is the primary structural element of plant cell walls and the most prevalent biological polymer naturally available.^77^ Because of their high absorbance properties, cellulose-derived cotton gauze dressings are utilized in therapeutic settings. These hydro fiber dressings work well for moderate to severe wounds because they react with wound exudates to form a soft gel.^78,79^ In a study of double-network hydrogels made with cellulose, Lu et al. demonstrated rapid coagulation and adhesiveness and exceptional antibacterial activity.^80^ During the in vivo study, these hydrogels enhanced tissue adhesion and hemostatic capacity and had superior action while also promoting blood coagulation through platelet and erythrocytes migration. In a similar study, Deng et al. used cellulose and fenugreek gum to create a composite hydrogel that was porous, absorbed a lot of water, and was thermally stable.^81^ Shefa et al. used a tempo-oxidized cellulose nanofiber (TOCN) to create a hydrogel system for the administration of curcumin, which produced neoepidermise and granulation of tissue at the wound site.^53^

### 2.5 Dextran-Based Hydrogels

Dextran is a biopolymer composed of glucose monomers with a α-1,6-linkage. Dextran facilitates the removal of toxic substances and exudates from wound sites, hence allowing faster healing. It shields the skin from ischemia damage and encourages the development of blood vessels. Wound healing and therapeutic neovascularization are two applications of dextran hydrogels.^82,83^ They could be used to treat skin infections and eliminate bacterial biofilms. Nevertheless, for efficient tissue regeneration and repair, dextran’s bioactivity is inadequate. To increase dextran’s medicinal potential, researchers have investigated oxidized and acetylated forms of the protein for quicker wound healing.

Cerium oxide and curcumin were incorporated in oxidized dextran and gelatin to generate a nanohybrid hydrogel.^25^ Significant anti-inflammatory and antioxidant qualities as well as prolonged drug release and improved cellular motility were demonstrated by the hydrogel. There is great promise for this hybrid system in wound dressing. Fu et al. demonstrated the exceptional antioxidative and antibacterial characteristics of oxidized hybrid hydrogels of chitosan with dextran loaded with polydopamine nanoparticles.^34^ To accelerate wound healing, these hydrogels could be used as a dressing. In another study, halloysite nanotubes of dextran and chitosan-doped sticky hydrogels were synthesized using a Schiff base reaction.^84^ Because of their inherent antibacterial qualities, these hydrogels demonstrated a significant bactericidal effect. These hydrogels greatly enhanced the healing of *S. aureus*-infected, methicillin-resistant wounds, making them appropriate for first-aid biomaterials for rapid hemostasis and infected wound restoration.^84^ Guan et al. investigated a gelatin/dextran-based hydrogel for wound healing.^35^ Excellent plasticity, injectability, blood coagulation capabilities, and self-restoration were demonstrated by this hydrogel. In a similar study, dextran-based apocynin-loaded hydrogel was found to decrease wound inflammation and accelerate skin tissue regeneration and angiogenesis in in vivo studies. This apocynin-loaded dextran hydrogel has promising future applicability in skin tissue regeneration and may be utilized for efficient wound healing.

### 2.6 Starch-Based Hydrogels

Starch is made of glycosidic macromolecules amylose and amylopectin and is the world’s second largest natural biomaterial. In therapeutic applications, it serves as a biomaterial for hemostasis.^85^ Mao et al. investigated gelatin and starch hydrogels with self-contracting property, which led to faster tissue repair and thicker layers of dermis and epidermis.^86^ In comparison to sutured wounds, these hydrogel-treated wounds had smoother skin and no discernible scarring. Owing to its exceptional efficacy, the hydrogel showed great promise as a noninvasive wound closure alternative to sutures used in medicine.^86^ Ahmed et al. synthesized hydrogel membranes for wound healing by combining silver-plated titania nanoparticles and graphitic carbon nitride with starch and poly(vinyl alcohol).^87^ The outcomes demonstrated full healing in 1 week, oxygen permeability, and extensive absorption of wound exudate.

### 2.7 Tragacanth Gum-Based Hydrogels

Tragacanth gum (TG), a renewable polysaccharide, is sourced from the plant species Astragalus. Wound healing is greatly influenced by the mineral content of TG, which contains calcium and magnesium. Calcium is necessary for cellular metabolism and thermoregulation of the skin in mammals, whereas magnesium facilitates the migration of keratinocytes and fibroblasts. Although TG lacks antibacterial qualities, it is commonly employed in hydrogel compositions to promote wound healing.^88,89^ It might be altered or functionalized to have a microbicidal effect or could be packed with medications for enhanced healing. Hemmatgir et al. used tragacanth gum/poly(vinyl alcohol) (PVA) and polyethylene glycol diacrylate (PGDA) to create a hydrogel for wound healing.^90^ In addition to being nontoxic, the hydrogel has outstanding antibacterial properties. Thus, TG-PA-PGDA hydrogel may be advantageous as a wound dressing.^90^ For pain and infection, tragacanth gum, PVA, and poly(vinylpyrrolidone) (PVP) were also used to generate hydrogels filled with lidocaine and gentamycin. The hydrogel composition showed the ability to gradually release medicine and absorb the wound exudate. The hydrogel dressings’ unique blend of mucoadhesiveness, antioxidant, and antibacterial qualities makes them an excellent substitute for treating wounds.^91^

### 2.8 Pectin-Based Hydrogels

Pectin, an anionic polymer produced by plants, is made up of galactose, galacturonic acid, xylose, fructose, rhamnose, and arabinose.^92^ Pectin is easily converted into hydrogel formulations that are helpful for wound dressings and preservation of acidic environments, both of which function as strong inhibitors of bacterial growth/infections. Pectin hydrogels resemble soft tissues, and the polymeric long-chain section promotes the growth and division of tissue cells. Electrostatic contact results from negatively charged functional groups such as carboxyl, which limit bacterial cell adhesion.^93,94^ Because pectin is hydrophilic, it reduces bacterial attachment and leaves the surface coated with hydration.

Giusto et al. created a pectin–honey hydrogel dressing, which demonstrates faster wound area reduction and overall healing.^95^ This suggests that applying the pectin–honey hydrogel helps wounds heal more quickly and effectively. Kocaaga et al. created hydrogel films of pectin with zeolite particles.^96^ This hydrogel showed an 86% drug release ratio in 5 h. The hydrogel formulation of the placebo demonstrated noncytotoxic properties while promoting cell migration and proliferation.^96^ In another study, for treating wounds infected with MRSA (methicillin-resistant *S. aureus*), pectin, HA (hyaluronic acid), and SA (salicylic acid) were used to make hydrogel films loaded with clindamycin (Cly).^97^ This formulated hydrogel showed strong antibacterial activity and drug release over a 12 h period.

### 2.9 Carrageenan-Based Hydrogels

The high molecular weight polysaccharide known as carrageenan (CRG) is derived from red seaweeds. κ-Carrageenan can be used for the production of thermoreversible gels with potential uses in the food and pharmaceutical industries.^52,98^ Because carrageenan hydrogels provide natural microenvironments that promote better interactions between cells and tissues, they are used to heal wounds. Carrageenan hydrogels, however, are less effective as wound dressings because of their poor mechanical performance, instability, and faster disintegration.^52^ To overcome these limitations, modifications in CRG hydrogels have been achieved by several techniques such as complexation, polymerization, and insertion of nanoparticles or biomolecules, resulting in an effective healing process.

For wound healing applications, hydrogel films consisting of sulfur nanoparticles, grapefruit seed extract, and κ-carrageenan have been created.^99^ The grapefruit seed extract helps in providing antimicrobial property against a wide range of bacteria, fungi, and molds. Additionally, it has antioxidant property which helps in faster tissue repair.^99^ The hydrogel film exhibited high swelling ratios, enhanced mechanical stability, and potent antibacterial properties. During in vivo studies, it was observed that the epidermal layer of the skin had fully healed. This hydrogel exhibits exceptional wound healing characteristics and has shown hemostatic capabilities in stressful medical settings.^99^ Poly(vinyl alcohol)/methyl kappa-carrageenan (κ-CaMA) hydrogels have a uniformly distributed symmetrical porosity and a dense, porous three-dimensional structure.^54^ These hydrogels have a large capacity for fluid retention as well as notable in vitro bioactivity. As observed from κ-CaC hydrogel, wound healing was successful in about 2 weeks in an in vivo model study. Histomorphological analysis showed fast epidermis remodeling and full re-epithelization.^54^ These results suggested that carrageenan hydrogels are a potential candidate for durable wound care and dressing material.

### 2.10 Xanthan Gum

Xanthan gum (XG), a natural polysaccharide, is derived from bacteria. It is an FDA-approved biopolymer and used as a stabilizer and thickener in food products, coupled with its role as a drug and protein carrier in nanomedicine, which underscores its versatility. Recently, it has also been used for production of hydrogels as it exhibits anti-inflammatory and wound healing properties.

In a study, aloe vera extract was incorporated in XG hydrogel, which showed effective antibacterial, wound healing, and anti-inflammatory effects.^100^ Similarly, aloe vera-loaded chitosan and XG hydrogels were developed for treatment of skin diseases.^100^ It accelerates wound closure by promoting epithelialization and collagen synthesis. In another study, a nanocomposite comprising bimetallic silver and magnesium oxide nanoparticles, aloe vera extract, and XG was developed.^101^ This formulation was shown to inhibit bacterial pathogens while stimulating wound healing processes. Tang et al. developed a xanthan gum (XG) and polyacrylamide hydrogel showing excellent mechanical properties.^102^ The formulated hydrogel showed high drug-loading capacity and good biocompatibility which promoted effective would healing.^102^ Reyes et al. developed a collagen- and XG-based hydrogel which showed effective inhibition of *E. coli*.^103^ The hydrogel further showed high proliferative activity of fibroblasts and monocytes with no significant toxicity. Thus, xanthan gum-derived hydrogels have wide applicability for wound healing and other biomedical applications.

## 3 Antifungal and Antiviral Applications of Hydrogel

Fungal keratitis is a significant threat to vision and can lead to blindness.^104^ Conventional treatment relies on topical antifungal eye drops due to their noninvasive and cost-effective nature.^105^ However, this approach suffers from limitations in corneal residence time (1–2 min) and poor penetration into the aqueous humor.^106^ To overcome this challenge, Chaves et al. developed a pH-responsive chitosan-based hydrogel formulation for oral administration.^107^ This system allows for the controlled release of dapsone and potentially reduces the required dosage while minimizing side effects. The pH responsiveness tailors drug release based on the varying pH conditions within the gastrointestinal tract. Similar hydrogel strategies can be explored for antifungal medications such as miconazole nitrate (MN) and itraconazole (ITR). ITR treats a broad spectrum of dermatophytes, e.g., *Microsporum*, *Epidermophyton*, and *Trichophyton* spp. MN, a broad-spectrum antifungal, exhibits limited water solubility, compromising its therapeutic effectiveness and employed as a topical treatment for dermatophytosis and superficial mycoses and as an oral gel for the treatment of *Candida* infections.^108^ Jain et al. successfully addressed this issue by formulating MN-loaded solid lipid nanoparticles (SLN) incorporated into a topical hydrogel, achieving sustained drug release.^108^ Likewise, ITR demonstrated improved antifungal activity when delivered via a topical hydrogel compared to commercially available therapies for tinea pedis.^109^ Sertaconazole (STZL), an imidazole antifungal used topically for dermatophytosis, has also been formulated into a hydrogel for enhanced treatment efficacy.^110^ Sahoo et al. developed a topical hydrogel containing STZL microemulsion (HSM) embedded in a Carbopol 940 base, leveraging the hydrophilic properties of Carbopol for optimal gel formulation, showing an effective therapeutic outcome.^110^

Acyclovir (ACV), a guanosine antiviral medication, remains a mainstay in the treatment of herpes simplex virus (HSV) infection. However, topical administration of ACV is hindered by its poor transdermal penetration and limited water solubility. Al-Subaie et al. designed an optimized ACV nanoemulsion hydrogel to overcome the shortcomings of commercially available ACV creams.^111^ Their formulation incorporated the optimized hydrogels into a nanoemulsion using chitosan, a biopolymer, as the gelling agent and Eugenol, a known skin permeation enhancer. Compared with the raw ACV hydrogel and commercially available ACV creams, the optimized ACV nanoemulsion hydrogel demonstrated significantly enhanced drug bioavailability. Tannic acid-modified silver nanoparticles (TA-AgNPs) have demonstrated promising antiviral properties against herpes simplex virus (HSV) by inhibiting viral attachment, penetration, and cell-to-cell spread.^112^ However, their liquid form hinders their localized application in HSV treatment. Szymanska et al. addressed this challenge by developing multifunctional mucoadhesive hydrogels.^113^ These hydrogels incorporate TA-AgNPs within a Carbopol 974P gel matrix, significantly inhibiting HSV-1 and blocking HSV-2 attachment and penetration. Furthermore, Houston et al. explored the virucidal activity of a pomegranate rind extract (PRE) and zinc sulfate (ZnSO_4_) combination against HSV-1, HSV-2, and even acyclovir-resistant strains of HSV-1.^114^ Based on these findings, they formulated a topical hydrogel delivery system containing 2.5% hydroxypropyl methylcellulose (HPMC), 1.25 mg/mL PRE, and 0.25 M ZnSO_4_ in a pH 4.5 phthalate buffer. This hydrogel aims to facilitate the topical delivery of the key bioactive components from PRE and Zn(II) across mucus membranes to target HSV clusters while preserving their virucidal and anti-inflammatory effects.

## 4 Hydrogels for Mucosal and Oral Infections

Periodontitis, which is the second most common oral illness, is a common oral condition affecting the gums, alveolar bone, and periodontal membrane.^115^ Chronic periodontitis is caused by microorganisms in tooth plaque, leading to an overflow of periodontal pus that can resorb alveolar bone and undermine tooth support.^116^ Severe cases may result in tooth loss or loosening.^117^ Research indicates a correlation between systemic disorders such as diabetes, cardiovascular disease, and Alzheimer’s disease and periodontitis.^118−120^ Tissue engineering technologies are used to treat periodontitis by achieving endogenous regeneration, reducing alveolar bone resorption, regenerating tissue, and controlling inflammation. Hydrogels, due to their scaffold structure and biocompatibility, are often used as biobased materials to regulate inflammation, alter the immune system, and serve as scaffolds for the regeneration of alveolar bone and periodontal tissue.^121^ One of the main causes of periodontitis is *Porphyromonas gingivalis*, as it invades periodontal tissues, forms plaque biofilms, and activates the immune system. The three primary stages of periodontal tissue regeneration are the inflammatory, restorative, and remodeling phases. Treatment strategies include managing the overabundance of pathogenic bacteria in the early stages, preventing the deterioration of periodontal pockets and alveolar bone resorption in severe cases, and encouraging the regeneration of periodontal tissue.

Liu et al. developed ZIF-8 (zeolite imidazole ester backbone 8) nanoparticles loaded with dexamethasone doped (DZIF) into a gelatin matrix to produce an injectable hydrogel with acid-responsive release, which effectively inhibited *P. gingivalis* bacteria.^122^ It has been demonstrated that DZIF nanoparticles decrease the expression of periodontal inflammatory markers and enhance the osteogenic differentiation of cells, indicating possible uses in the proliferation, differentiation, and restoration of alveolar bone in periodontitis.

**Figure 3:**
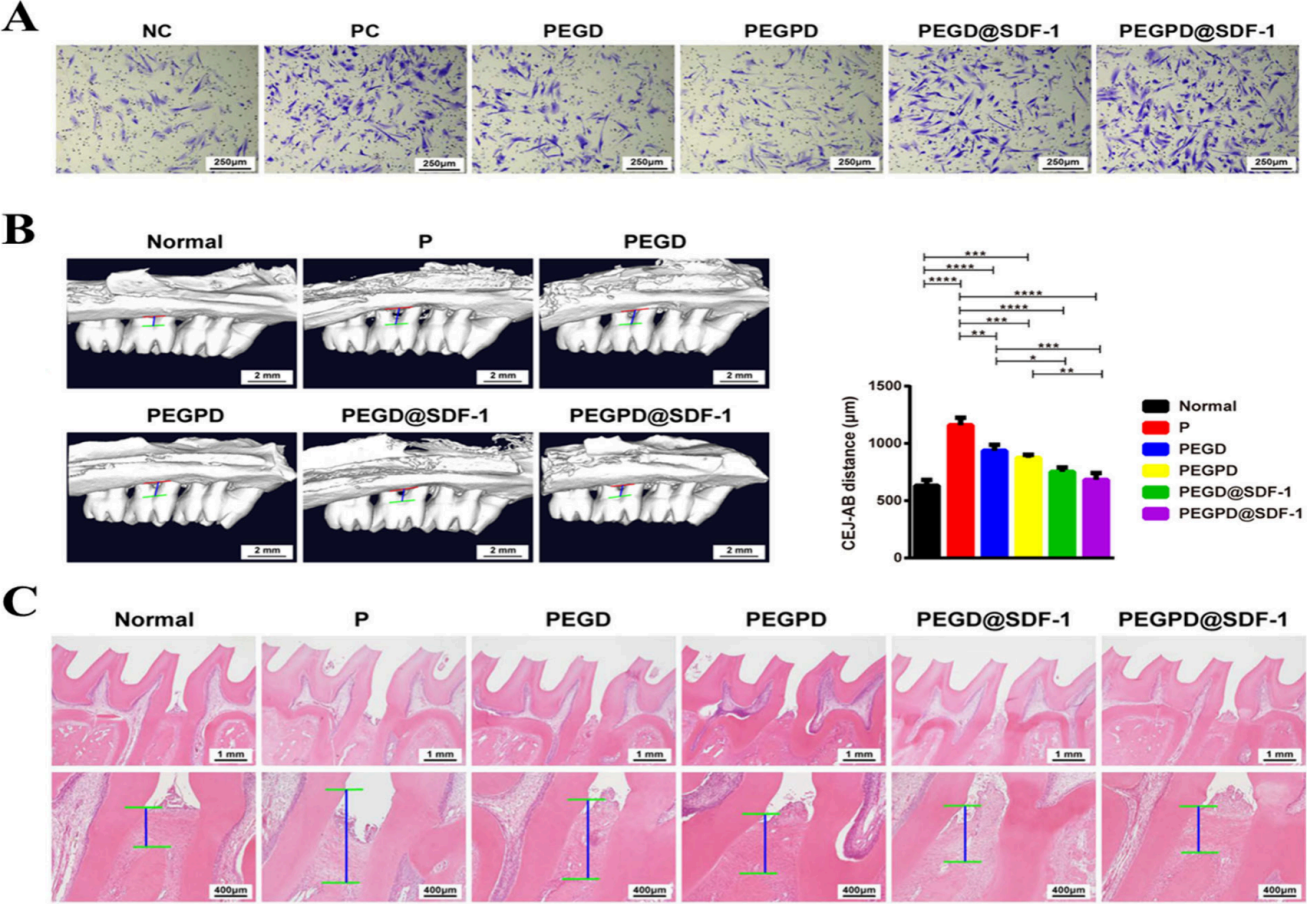
(A) Cell migration assay using the Transwell chamber showing gingipain-responsive hydrogel having efficient cell migration. (B) Three-dimensional reconstructed images of micro-CT and measurement of alveolar bone absorption 4 weeks after hydrogel injection showing PEGPD@SDF-1 with the most potent activity. Data are presented as mean ± SD, *N* = 6. *, *p* < 0.05; **, *p* < 0.01; ***, *p* < 0.001; ****, *p* < 0.0001. (C) H&E staining images of periodontal tissue between the maxillary first molar and the second molar, 4 weeks after hydrogel injection. Adapted with permission from ref (31). Copyright 2021 American Chemical Society

Liu et al. developed a peptide-based hydrogel that exhibited an enzyme-responsive release.^31^ This antimicrobial peptide was found to have anchoring peptides that were cleaved by Rgp, triggering SAMP release upon contact with *P. gingivalis* (Figure 3). Mehra et al. developed soy protein-based hydrogel encapsulated with antimicrobial drug ciprofloxacin.^123^ This hydrogel demonstrated a controlled release of the drug with no cytotoxicity. Further, this soy–hydrogel demonstrated significant antibacterial activity in the oral cavity. In a similar study against chronic periodontitis, Zhao et al. coupled metformin and doxycycline, two antimicrobial drugs, on a hydrogel to improve the loading efficiency of the drug and triggered release in reactive oxygen species.^26^ When combined with beta glycerol phosphate, chitosan’s antimicrobial qualities can create a temperature-sensitive hydrogel that goes through a sol–gel transition at physiological pH and temperature. Xu et al. have shown that loading aspirin along with erythropoietin in a chitosan hydrogel can effectively terminate inflammation and restore alveolar bone height.^3^ Additionally, adding lipoxin A4 and doxorubicin (DOX) in the chitosan hydrogel reduced gingival bacterial infection.^124^

Controlling chemotaxis and polarizing immune cells to get anti-inflammatory response is a good therapeutic strategy against *P. gingivalis* oral infection. The 3D structure of hydrogels regulates a range of proinflammatory cytokines to efficiently lower the damage to periodontal tissues and elevate cell differentiation and multiplication by loading various growth factors and immune modulators. This provides basic conditions for subsequent regrowth of periodontal tissues.^125^ Hydrogels, which reduce inflammation and adjust the immune system, are used to treat periodontitis. Dental pulp stem cells, due to their immunomodulatory properties, are used for anti-inflammatory treatments like periodontitis.^126^ Their exosomes control phagocyte phenotype, making them beneficial.^127^ Their performance boosts anti-inflammatory phagocytic percentage, accelerates bone regeneration, and promotes immune system modulation, ultimately curing periodontitis.^128^

Reactive oxygen species (ROS) can upset the oxidative equilibrium and intensify inflammation when they are released during the host immunological response.^129^ Bone and periodontal tissue regeneration can be aided by regulating ROS levels. Potential ROS scavengers dithiothreitol (DTT) and stromal cell-derived factor-1 (SDF-1) promote cell division and proliferation.^130^ To explore their synergistic impact, a temperature-sensitive hydrogel was developed by Liu et al. using DTT and SDF-1 to study the activity against periodontitis.^131^ This hydrogel showed excellent antibacterial activity, which aided the periodontal tissue regeneration.

Multiple studies have demonstrated the biocompatibility and prospective use of chitosan as an injectable biohydrogel-loaded therapeutic or scaffold for tissue engineering, making it a useful temperature-sensitive smart hydrogel. Zang et al. improved periodontal tissue by using ornidazole and bone morphogenetic protein-7 coloaded with chitosan hydrogel to increase human periodontal membrane fibroblast adhesion, decrease connective tissue, and promote osteogenesis as studied in vivo in class III root fracture defects, making it suited for severe periodontitis patients.^132^ Srinivasan et al. used nanobioactive glass ceramic and PLGA to build a three-layer nanocomposite hydrogel.^133^ Each layer was loaded with biomolecules that encouraged the development of cells in the tissues. The composite effectively sealed flaws, showcasing a novel approach to periodontitis therapy.

### 4.1 Small Molecules Delivery

Small molecules are low molecular weight compounds typically produced synthetically. Oral distribution of anti-inflammatory drugs can lead to adverse reactions (e.g., immunosuppression and hepatotoxicity) because of systemic exposure. Because hydrogel-based administration technologies concentrate the therapeutic payload locally inside mucosal tissues, they may significantly reduce inflammatory disorders such as inflammatory bowel disease (IBD).^134^ Ascorbyl palmitate (AP) hydrogels can target inflammation and are naturally amphiphilic, which makes them useful for incorporating anti-inflammatory drugs that are lipophilic. Because of its amphiphilicity, lipophilic dexamethasone may be encapsulated and micelles can assemble themselves into a microfiber hydrogel. In the in vivo model, these hydrogels were found to stick to the inflammatory mucosa of the intestine with slow and sustained release of the drug significantly minimizing the infection.^134^ Similar approaches have been utilized for the buccal distribution of dexamethasone-loaded hydrogels.^135^ The lipophilic corticosteroid drug “mometasone” has been encapsulated in thermosensitive microspheres of copolymer hydrogel and injected into the sinonasal region. The thermoresponsive nature permitted the hydrogel to adapt to the intricate anatomy of the sinonasal region in advance of body temperature gelation and mucosal layer adhesion. A rabbit model of chronic rhinosinusitis showed decreased inflammation after intervention with mometasone-loaded hydrogels.^136^ Thus, these findings show that lipophilic medicines with low bioavailability and/or higher metabolic elimination may be appropriately administered to specific areas using amphiphilic hydrogels.

In another work, clarithromycin (CAM) was effectively packed into liposomes by Chen et al., and these liposomes were subsequently encapsulated in a hydrogel of polyethylene glycol.^137^ Acute bacterial rhinosinusitis in the rabbit model was used to study the efficacy of the injectable CAM–liposome-laden PEG hydrogels. Inhibition of *S. pneumoniae* was observed with no signs of adverse reaction and infection. Antibiotics have also been administered via hydrogels to combat dental periodontitis.^42,138^ In an interesting work, Wroblewská et al. studied the effectiveness of metronidazole encapsulated in a hydrogel made of alginate.^42^ The results showed that alginate hydrogels had excellent antibacterial properties, cumulative permeation into the porcine buccal mucosa, and extended mucosa retention.^42^ Antifungals have also been developed to be administered to mucosal areas by using hydrogels. Senel et al. developed a hydrogel of chitosan encapsulated with chlorhexidine gluconate to study activity against infection of the oral cavity caused by *Candida albicans*.^43^ This hydrogel had strong in vitro antifungal efficacy toward *C. albicans* and reached a drug retention duration exceeding 3 h. Mirza et al. developed itraconazole lipidic nanoparticles loaded into a hydrogel made of Pluronic F to eliminate *C. albicans* in the vaginal canal.^139^ In a disease model of *C. albicans* in rats, the hydrogel compositions maintained microbicidal efficacy for 21 days, outperforming a widely accessible antimicrobial ointment (Gynazole).

### 4.2 Delivery of Biologics

Biologics are complex molecules produced by living cells naturally or with the aid of biotechnological methods. With the advancements in genetic engineering and recombinant protein manufacturing in recent times, the use of biological procedures, such as monoclonal antibodies, has rapidly increased. But, to combat conditions like IBD, parenterally delivered biologics frequently display an extremely low bioavailability at the mucosal tissues, necessitating the administration of high dosages. The latest research indicates that in cases when novel intranasal administration methods are preferred, mucosal vaccination administration may provide larger preventive advantages. This encourages the utilization of hydrogels for transferring glycan, protein, or nucleic acid medications straight away to the mucosal surface, enabling minimally invasive therapies. Hydrogel carriers can offer further protection against the quick enzymatic breakdown of biological contents when administered. In earlier research, heparin, a naturally existing glycosaminoglycan and anticoagulant, was incorporated into hydrogels to treat IBD locally. In a rat model of colitis, heparin was administered after incorporation into an injectable hydrogel made of bovine serum albumin. It was observed that this hydrogel showed selective attachment to inflammatory tissue and local release of heparin as an outcome of its net-negative charge. This prevented inflammation and microthrombosis, which further inhibited the IBD progress. Varma et al. developed intranasal gel to deliver the subunit of influenza vaccination.^140^ Using a dual-barrel syringe, intranasal gel precursors comprising dextran and branched polyethylenimine (PEI) were delivered. Long-term mucoadhesion and nasal retention have been demonstrated using this dextran–PEI hydrogel, which resulted in strong mucosal immune reactions.^140^ A similar investigation was carried out by Bedford et al. to investigate the efficacy of the vaccine-encapsulated hydrogel of chitosan in protecting mice against infection with influenza.^141^ Mice that were intranasally immunized with chitosan hydrogel that had been injected with antigens related to influenza showed strong mucosal immune reactions as well as defense against influenza virus.^141^ Hydrogels have also been utilized for gene therapy in the application of ocular wound healing. In this work, a chitosan-based in situ gelling technique was used to administer Connexin antisense oligonucleotide (ASON) to rats following ocular impairment. This investigation proved that the ASON was successfully delivered into the damaged tissue.^41^ To enhance mucosal gene therapy, genes can also be encapsulated with a hydrogel with liposomes or polyplex nanoparticles.^142^

## 5 Hydrogels for Cancer Immunotherapies

Injectable hydrogels (IHs) have emerged as promising platforms for cancer immunotherapy due to their ability to deliver therapeutic agents directly to tumor sites. The application of injectable hydrogels is rapidly expanding in various modalities of cancer immunotherapy, including radioimmunotherapy, photothermal immunotherapy, and photodynamic immunotherapy. These hydrogels serve as versatile carriers for delivering immune-stimulating agents, radioisotopes, photothermal agents, and photosensitizers, enhancing antitumor immune responses while minimizing systemic toxicity. Through localized delivery and controlled release, injectable hydrogels offer a targeted approach to augmenting the efficacy of cancer immunotherapy across multiple treatment modalities (Table 1).

**Table 1:** List of Injectable Hydrogels (IHs) Used for Cancer Treatment Using Various Therapeutic Agents^a^

types	IHs	therapeutic agents	model of tumor cells	ref
chemoimmunotherapy	MRD peptide	melittin and DOX	B16F10 melanoma	(144)
	PEGSH and PEGDA	DOX and R837	B16F10 melanoma	(145)
	PLN-PEG	DOX and l-norvaline	B16F10 melanoma	(146)
	HAMA, UPyMA, and DEGMA	PD-L1 antagonist d-peptide and DOX	CT26 colorectal carcinoma	(147)
	PELG-PEG-PELG	DOX, IFN-γ, and IL-2	B16F10 melanoma xenograft	(148)
	PEG-P“Glu”	αPDL-1 antibody and Zeb	B16F10 melanoma	(5)
	PEG and γ PEG	cisplatin and IL-15	B16F0-RFP	(149)
	CpG DNA	DOX and CpG	colon-26 colorectal cancer	(150)
	α-CD/PEG-4000	DOX and CpG	B16 melanoma	(151)
	α-CD and PEG-4000	PEI-CA-DOX, DCs, and CpG	B16 melanoma	(152)
	diCPT-iRGD/CDA-NT	STING agonist cyclic di-AMP and camptothecin	GL-261-Luc brain, 4T1-Luc breast, and CT26 colon cancer cells	(153)
radioimmunotherapy	alginate–Ca^2+^	^131^I-Cat and CpG	CT26 colorectal carcinoma, 4T1 mammary carcinoma, rabbit VX2 liver cancer, and prostate cancer PDX	(154)
	PETyr	^131^I and SmacN7-R9	HepG2-Luc tumor	(155)
	aptamer-conjugated alginate	CpG-cAptamer and oxaliplatin	CT26 colorectal carcinoma and 4T1 mammary carcinoma	(9)
	Smac-TLR7/8	Smac N7 peptide and toll-like receptor agonist TLR7/8a	B16 melanoma	(156)
	hydroxy propyl cellulose	X-ray irradiation and IFN-α2b	MKN-45 human gastric carcinoma	(157)
photothermal immunotherapy	genipin-cross-linked CpG NP-loaded PEG-IR-820-α-CD	CpG and IR820	B16 melanoma	(158)
	hexapodna	CpG sequences and gold NPs	EG7-OVA tumor-bearing mice	(159)
	PLEL	ICG, R848, and CPG ODNs	4T1-Luc breast cancer	(160)
	TIMmH	SPIIN and CpG	4T1 breast cancer	(161)
	GG	POM and R848	4T1 mammary carcinoma	(162)
	PND	MnO_2_	4T1 mammary carcinoma	(163)
photodynamic immunotherapy	PEGDA	R837-loaded PLGA NPs and Ce6-Cat	CT26 colorectal and 4T1 mammary carcinoma	(164)
	alginate–Ca^2+^	Ce6 and R837	4T1 mammary carcinoma	(38)
	PF-PEG NPs	Ce6 and NLG919	4T1 murine breast cancer	(165)
	FK-PBA	PEI-Ce6	murine CT26 colorectal, B16-OVA, and B16-F10-Luc melanoma cancer cells	(166)

a Adapted with permission from ref (143). Copyright 2023 Acta Materialia Inc. Published by Elsevier Ltd.

### 5.1 Injectable Hydrogels for Chemoimmunotherapy

**Figure 4:**
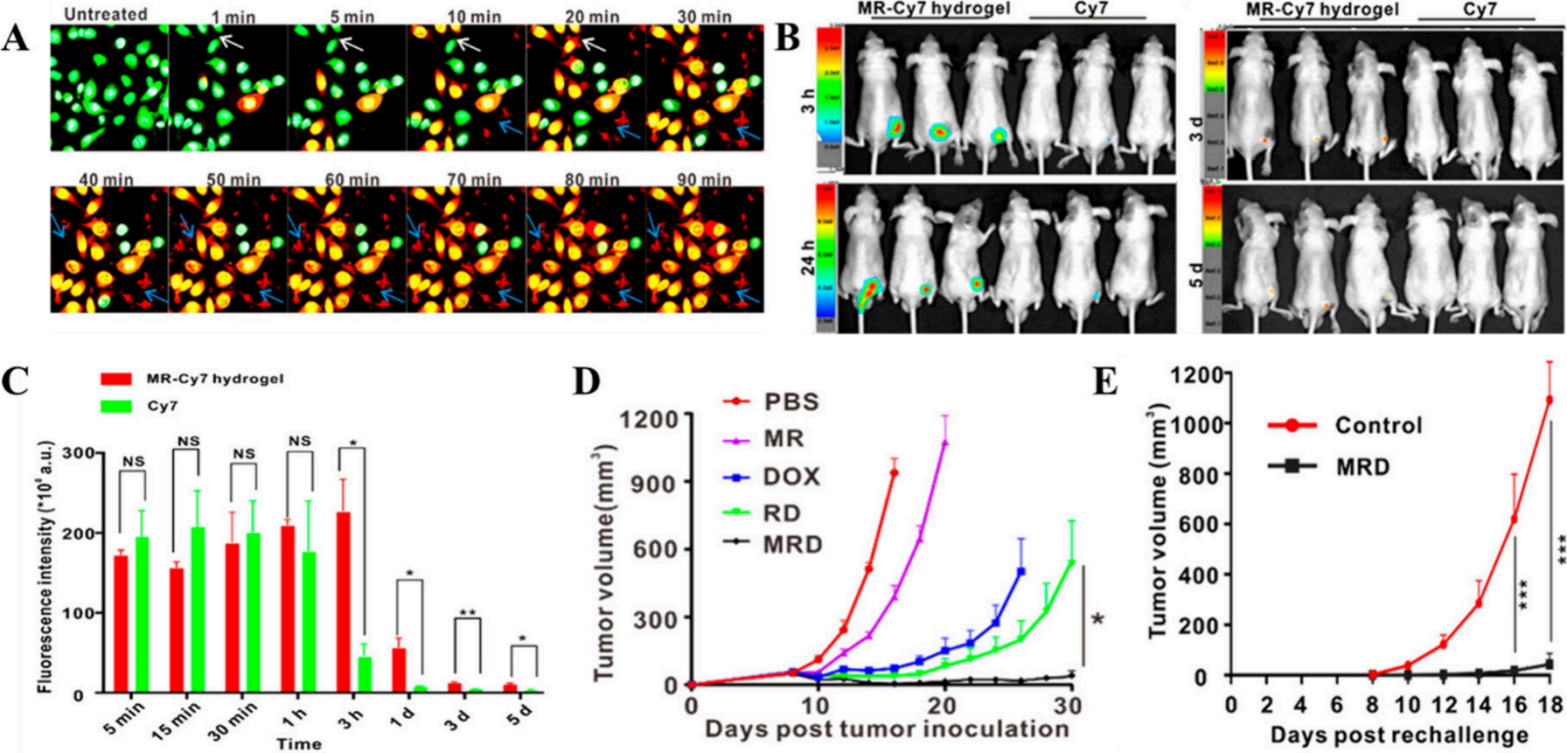
(A) Confocal real-time fluorescence images of B16-GFP cells treated with the melittin-RADA32-Dox (MR-Dox) hydrogel. For estimating cell death, propidium iodide (red) was used. Untreated cells showed green fluorescence, while with increasing MR hydrogel treatment duration, cells showed yellow fluorescence (red + green), indicating increased cell death. (B) In vivo NIR fluorescence imaging of the distribution of MR-Cy7 hydrogel and Cy7 dye after subcutaneous injection at various time points (C) along with its quantitative data showing more cy7 accumulation in the case of hydrogel-mediated delivery for a prolonged period. (D) Tumor growth curves for mice intratumorally injected with PBS, DOX, RD, MR, or MRD hydrogel. (E) Quantification of the second tumor growth showing long-term immune-memory effects of MRD hydrogel. Adapted with permission from ref (144). Copyright 2018 American Chemical Society.

The efficacy and outcome of chemotherapy are typically restricted by the rapid elimination of the drugs and off-target toxicity such as liver dysfunction, myelosuppression, neurotoxicity, cardiotoxicity, etc. Injectable hydrogels (IHs), which can locally deliver cytotoxic chemotherapy medicines, may provide advantages over traditional chemotherapy for long-lasting and effective care.^167,168^ Multiple researchers have shown that several kinds of chemotherapy drugs, including mitoxantrone and anthracycline family drugs paclitaxel and docetaxel, may stimulate immunological cell death (ICD) in tumor cells.^169^ This, in turn, may lead to the generation of cytokines and damage-associated molecular patterns (DAMPs), which, in turn, may promote adaptive immunity. Additionally, therapeutic efficacy is severely hampered by tumor microenvironments (TMEs). Jin et al. developed peptide nanofibers encapsulated with chemotherapeutic drugs and melittin.^144^ The chemotherapeutic drug doxorubicin is a common inducer of ICD. A cationic polypeptide “melittin” can destroy TAMs that mimic M2 macrophages. However, the hemolytic activity of melittin often restricts its in vivo use. To overcome this limitation and increase the effectiveness of therapy, cationic melittin peptides and synthetic amphiphilic RADA32 were combined via the GG linker to generate a hydrogel encapsulated with doxorubicin and melittin (Figure 4). Compared to treatment with hydrogel and DOX, melanoma cancer cells treated with doxorubicin–melittin hydrogel showed 5–6 times higher cell death. Furthermore, the melanoma cells administered with the doxorubicin–melittin hydrogel showed a reduction in the S phase of the cell cycle and inhibition at the G2/M phase. The uniform distribution and increased bioavailability in the TME caused tumor reduction by around 95%. The MRD IHs’ slow and sustained release of melittin significantly decreased the number of immunosuppressive macrophages. Further studies on the mechanism of tumor recurrence indicate that the MRD IHs had fully acquired robust immunity and long-term memory. Increasing dendritic cell (DC) maturation and stimulation in tumor cells is another alternative to increase anticancer immune responses.^170^ Li et al. developed an injectable hydrogel composed of PEG-thiol and PEG-diacrylate loaded with doxorubicin and imiquimod for synergistic melanoma therapy.^145^ Based on their findings, the combination drug released from the IHs promoted both apoptotic and nonapoptotic death of cells. From in vivo studies, it was observed that tumors and metastatic development were significantly inhibited by the drug-released immune response amplification through dendritic cell (DC) development, M1 macrophage recruitment, interferon γ, and tumor necrosis factors-α release. The prolonged release of doxorubicin and imiquimod in response to IHs was an efficient chemotherapy approach as it decreased immunosuppressive responses such as IL-10 production and M2 macrophage infiltration. It has also been shown that immune checkpoint inhibitor (ICI)-deficient tumor immune microenvironments may reactivate suppressed T-cell-mediated immunity. ARG1 pathway blocker hydrogel was developed by Ren et al. using l-norvaline and doxorubicin-coencapsulated polypeptide block copolymer.^146^ After being administered to the tumor locations, the block copolymer mixture soon changed into a hydrogel that delivered l-valine gradually while also releasing DOX due to the action of peptide breakdown enzymes. During an in vivo study on the B16F10 mouse model, it was demonstrated that ICD produced by doxorubicin in conjunction with the ARG1 immunosuppressive environment enhances the development of DCs. This further promoted systemic immunity, reduced the development of tumor growth, and caused lymphocyte internalization and aggregation within the tumors. According to this study, this immunoregulatory IH mechanism enhanced the efficacy of immunotherapy and supported TME for immunosuppression.^146^

ICI, when combined with cytotoxic chemotherapy drugs that target cancer cells directly, stimulates synergistic anticancer immune responses. Liu et al. developed an IH-containing antagonist peptide against PD-L1 and doxorubicin.^147^ It was concluded from their study that IH was quickly formed by hydrophobic interactions and hydrogen bonding, which subsequently released doxorubicin, which killed tumor cells, and DPPA1, which enhanced T-cell immunity and inhibited the PD-L1 pathway to promote anticancer activity. This enhanced cytotoxic T-cell tumor infiltration and stimulated extremely high levels of IFN-γ and TNF-α production. In the colorectal cancer model of mice, a synergistic tumor inhibition impact was observed, which further showed the potential to raise the level of response to immunotherapy and lower overall adverse effects. Similarly, a reactive oxygen species (ROS) active IH scaffold was developed by Wang et al. by encapsulating gemcitabine (GEM) and antibody against αPDL-1.^171^ From an in vivo study, the hydrogel was shown to enhance tumor inhibition driven by immune cells with no further tumor recurrence by the development of memory T cells and systemic tumor suppression. In another study, pH- and ROS-responsive IHs were developed by Ruan et al. for the codelivery of zebularine (Zeb) and PDL-1 antibody.^5^ At acidic pH in the tumor microenvironment and under ROS, Zeb and PDL-1 antibodies were shown to have a controlled release from this hydrogel. Studies on B16F10 melanoma mice revealed that the combination treatment decreased the development of tumors and increased their survivability. It was found from the study that when Zeb is released, it causes an anticancer immune response, boosts PDL-1 expression, reverses the immunosuppressive TME, alters tumor-associated antigen (TAA) expression, and releases αPDL-1.

For codelivery of interleukin-15 and cisplatin, Hu et al. developed thermosensitive IHs which are made of PEG and γ PEG di-block copolymers for cancer therapy.^149^ C57BL/6 mice bearing melanoma showed decreased tumor volume and weight with immune activity after administration of the coloaded IHs. Among the study group, the codelivery had the maximum impact on tumor growth inhibition with a tumor suppression rate of 88.8% after 18 days.

Dong et al. developed IHs for the codelivery of immune adjuvant and DOX with dual fluorescence imaging properties.^151^ The release, distribution, and metabolism of DOX and the immune adjuvant were evaluated by fluorescence imaging. Treatment resulted in positive therapeutic outcomes, as evidenced by a decline in tumor-associated macrophages and a spike in cytotoxic T cells (CD8+T). Prolonged immunological engagement was generated by the gradual breakdown of immune adjuvant, while the rapid release of DOX was observed at acidic pH.

### 5.2 Injectable Hydrogel for Local Radioimmunotherapy

To eradicate malignancies, radiotherapy (RT) employing radioisotopes or external ionizing radiation is commonly used in clinical settings. Integrating immunotherapy with radiation therapy can effectively increase the local effectiveness of RT and its ability to eradicate distant cancers^154^ The combination of ICI and RT has demonstrated effectiveness in treating tumors, such as melanoma and breast cancer.^172,173^ However, the systemic administration of radioisotopes may result in damage to healthy tissues and organs. Integrating immunotherapy with RT using hydrogels can significantly lower the adverse effects, providing a local drug administration platform.

Chao et al. developed a unique therapeutic hydrogel-based solution technique to address the drawbacks of traditional RT.^154^ They created an IH made of algae (ALG) that was filled with catalase loaded with radioisotopes ^131^I-Cat (^131^ICat/CpG/ALG). The goal of this approach was to start effective radiation therapy while also having immunotherapeutic efficacy.^154^ Interestingly, pure ^131^I and ^131^I-Cat showed only minimal retention and rapid elimination after 48 h post injection, but the ^131^I-Cat/ALG formulation showed strong retention inside the tumor.^154^ Treatment with ^131^ICat/CpG/ALG significantly reduced tumor metastasis, which successfully verified the strong therapeutic benefits of the developed hybrid hydrogel-based compositions of immunostimulatory drugs and radioimmunotherapy. Combination immunotherapy employing IHs may decrease ITM and boost immunity toward the tumor to counteract radioresistance.^156^ Liu et al. developed a transparent, porous, nanofibrous-structured IH and conjugated (Smac-toll-like receptor 7/8) Smac-TLR7/8 self-assembled peptide.^155^ The hydrogel was loaded with Smac mimetic peptide with a pro-apoptosis property to increase tumor radiosensitivity to low radiation doses.^155^ A hydrogel platform effectively controlled the repolarization of tumor-suppressing M1 macrophages under repeated γ-radiation. Tumor growth was successfully inhibited by this enhanced antitumor immunity and cytokine sequestration including TNF-α and IFN-γ. Tumor development was inhibited by treatments with free peptide and radiation Smac hydrogel by 11.0% and 57.2%, respectively. On the other hand, because of the synergistic effect of radioimmunotherapy, the Smac hydrogel and radiation association showed the highest tumor growth inhibition by 86%. M1 macrophages were much more prevalent than M2 in the synergistic treated group.^155^ A significant amount of proinflammatory cytokines linked to antitumor immunity was released by M1 macrophages. Significantly, these results demonstrate that the repolarization of tumor-associated macrophages can activate antitumor immunity and overcome the radioimmunotherapy-based drug administration system’s resistance to treatment.^155^ A further investigation assessed the application of immunotherapy in conjunction with antitumor treatment, including intraperitoneal T-cell injection and low-level X-ray exposure utilizing a hydrogel loaded with IFN-α2b. Tumors were first exposed to low-dose X-ray radiation before being injected with T lymphocytes. Finally, the hydrogel accelerated T-cell activation, destroyed tumor cells, and gradually produced IFN-α2b. Simultaneously, low-dose irradiation in tumors promoted IFNα-2b activation of T-cell proliferation and increased IFN-γ production.

### 5.3 Injectable Hydrogel for Local Photothermal Immunotherapy

One of the most recent approaches for treating cancer is photothermal immunotherapy (PTT). This method employs the transformation of near-infrared (NIR) wavelengths of light into heat to kill cancerous cells.^158^ Although PTT has demonstrated several benefits over traditional cancer treatments, it still has drawbacks, such as increasing its bioavailability in tumor tissue, off-target cytotoxicity, etc. To overcome these drawbacks, scientists are looking at the possibility of integrating PTT with other forms of therapy.

IR820 is a biocompatible, near-infrared fluorescent dye that is used for PTT of subcutaneous tumors and in vivo fluorescence scanning.^174^ Yata et al. investigated the combination of the PTT technique and immunostimulatory DNA hydrogel for tumor immunotherapy.^159^ The hydrogel was created by combining gold nanoparticles with DNA constructs containing CpG sequences. When the hydrogel was exposed to laser light, it generated hexapod-like DNA, which caused immune cells to secrete cytokines that promote inflammation. From the in vivo study, it was observed that exposure to hydrogel and PTT raised the local temperature, induced heat shock protein 70 mRNA expression, and raised specific IgG serum levels with increased tumor-associated antigen. This process demonstrated the prospect of integrating immunotherapy with PTT for inhibiting tumor development and prolonging the survivability of mice. In a similar study, an injectable hydrogel combining immunotherapy with PTT was found to prevent breast cancer relapse.^160^ Self-assembled multifunctional nanoparticles (RIC NPs), TLR antagonists, and CPG ODNs were combined to form a thermosensitive hydrogel. The immune components that are produced when RIC NPs are introduced into the resection cavity for PTT serve as a vaccine against postsurgical cancer immunotherapy (CIT). The findings indicate that this strategy may prevent metastasis and the recurrences of breast cancer. Dong et al. have created an efficient synergistic PTT system for cancer by IR820 conjugated hydrogel with NP-loaded and self-cross-linked by CpG DNA sequence.^158^ The engineered IHs can eliminate the primary tumor through hyperthermia and generate photothermally activated tumor antigens to support CIT. By inducing targeted antitumor immunity, as examined from immune cells inside the tumor microenvironment, this combination strategy showed a better systemic therapeutic impact than individual therapies such as PTT or immunotherapy. Additionally, PTT offers new opportunities for targeted treatment of cancer.^158^

Polyoxometalate (POM) is a photothermal substance that kills tumor cells by transforming light into heat. It has several benefits, such as biocompatibility, high photothermal conversion efficiencies, and durability.^175,176^ POM is evenly dispersed and has self-healing qualities in porous hydrogel networks. When POM was encapsulated in hydrogels, it showed a synergistic anticancer impact on 4T1 breast cancer, leading to a significant reduction in tumor development inhibition and a minimum amount of lung metastasis. Researchers have investigated the efficacy of an alternative PTT using gellan gum-based hydrogel loaded with POM to overcome some of the problems associated with conventional PTT and immunotherapy, such as toxicity and durability.^162^ Based on the PTT response, it was concluded that POM could successfully inhibit tumor growth. As was previously mentioned, this hydrogel administration strategy has been effective in providing high encapsulation of therapeutics with sustained release over a longer duration. The hydrogel approach in this investigation was successful in reducing systemic toxicities while enhancing PTT-based chemotherapy and antitumor immunity.

### 5.4 Injectable Hydrogel for Local Photodynamic Immunotherapy

Using light-activated molecules, photodynamic therapy (PDT) treats several diseases, such as antibiotic-resistant infections, cancer, and age-related macular degeneration.^177,178^ Even though photodynamic therapy (PDT) is an efficient anticancer treatment, its application is limited due to skin photosensitivity and issues with the solubility of phototherapeutic chemicals. Research in photodynamic cancer therapy has expanded with the development of IHs that can carry photosensitizers into tumors with sustained release.

Numerous investigations have shown that the hydrogel platform frequently generates adequate immune responses and can overcome existing PDT limitations. For instance, the effectiveness of PDT toward cancers is restricted by oxygen-deficient hypoxic TME. Meng et al. created a light-induced hydrogel scaffold in conjunction with catalase (CAT), immunological adjuvant R837, and PDT agent chlorin e6 (Ce6). After in vivo administration and exposure to light (660 nm), ROS was produced by chlorin e6 which showed antitumor activity.^164^ The findings of this study suggest that light-activated in situ gelation stimulation with only one injection can greatly enhance photoimmunotherapy.

Shu et al. enhanced immunotherapy over tumors by developing a revised photodynamic immunotherapy (PDIT) approach employing Ce6 with R837 adjuvant loaded in hydrogel.^38^ It has been observed that this PDIT-activated hydrogel resulted in dendritic cell maturation, which further accelerated immune response against the tumor cells. In a similar study, an alginate-based PDIT hydrogel loaded with R837, TLR7, and Ce6 was studied in a breast cancer in vivo model. After intraperitoneal administration, photodynamic therapy was induced. With this approach, promising treatment outcomes in the 4T1 breast cancer mouse model were observed. Although a systemic anticancer immune response is triggered by PDT, multiple PDT doses must be administered to sustain a significant antitumor immune response.^179^ To overcome this restriction, PDT is driven using the luminescence from persistent luminescent materials (PLM) naturally through a broad range of excitation energy. PLM could receive excitation energy from several sources of light such as near-infrared (NIR), X-ray, ultraviolet, and, visible light.^180,181^ A rechargeable immune IH was developed with R837, PLM, and excess calcium ions. Because of the uniform nature of these IHs, PLM can uniformly distribute through the material. In vitro and in vivo studies indicated that dendritic cells were activated post administration of the PDIT hydrogel, which led to the production of IL-6- and TNF-α-induced tumor killing. Xing et al. developed a PDIT hydrogel with polymeric NPs encapsulated with Ce6 and indoleamine 2,3-dioxygenase (IDO) inhibitor (NLG919). Using fluoridated polymers, this approach increases the efficacy of photodynamic therapy.^165^ The administration of this hydrogel showed synergistic PDIT activity on the tumors. Fang et al. produced a PDT-activated autologous tumor cell vaccine.^166^ The vaccine consists of a hydrogel that was developed by dialysis and physical blending. In colorectal and melanoma cancer model in vivo, it has been discovered that a combination of tailored IH and PDT suppresses regulatory T cells, strengthens neoepitope-specific CD8+ T cells, and prevents tumor recurrence with no obvious systemic side effects.

## 6 Hydrogels for Tissue Regeneration

The ability to inject minimally invasively, localize and then heal autonomously, and restore mechanical properties makes self-healing injectable hydrogels a promising class of biomaterials at the forefront of tissue engineering strategies. In a medical conditions like myocardial infarction, brain damage, or a vitrectomy, the self-healing hydrogels’ mechanical support is adequate to encourage tissue regeneration.^33,182^ It should be noted that the rheological properties of injectable hydrogels may be altered when administered via an extended percutaneous catheter in contrast to a tiny syringe.^183^ Lopez Hernandez et al. studied the comparative efficacy of hydrogel delivery using syringes vs catheters.^183^ This study provided statistical boundary values for consistency index (*k*) and flow behavior index (*n*) for both delivery modes. Another advantage of self-healing injectable hydrogels is that they can be customized based on patient-specific tissue abnormalities. The utilization of noninvasive imaging modalities in fusion with injectable hydrogels enables real-time optimization of hydrogel volume, distribution, and mechanical properties to achieve patient-specific healing strategies.^184^ Bioactives could be encapsulated into self-healing hydrogels to help with tissue regeneration or to provide medications to treat illnesses. Encapsulation of medications and rapid self-healing enables the hydrogel to increase its effectiveness while reducing side effects.^185^ One challenge of injected hydrogels is their retention at the injection site and their biological distribution. In a recent study, Schotman et al. highlighted the significance of administered volume, hydrogel mechanical characteristics, and biological variables like hydrogel-tissue affinity and tissue contractions.^185^ Drug size, solubility, and interactions with the hydrogel matrix affect the drug release kinetics from hydrogels.^186^

Hydrogels for tissue regeneration are often engineered to create the ideal physicochemical environment for cell ingrowth and subsequent tissue regeneration due to factors such as cell adhesion ligands, porosity, and viscoelasticity.^187,188^ Therefore, cell encapsulation therapy, which involves delivering genetically modified cells that secrete reconstructive growth factors or the administration of live cells or progenitor cells with hydrogels, can support tissue regeneration.^189^ The introduction and maturation of local cells are greatly impacted by the mechanical characteristics of the hydrogels. For example, it has been observed that adipogenesis is stimulated by smooth hydrogels, but stem cell osteogenesis is induced by solid hydrogels.^190^ Several injectable hydrogels are under clinical trials composed of self-healing abilities that can be used in a variety of ways to promote tissue regeneration. They can be used for several applications such as hydrogels to provide mechanical support or controlled distribution of drugs or cells for cardiac tissue regeneration, spinal cord injury, bone regeneration, etc.

### 6.1 Cardiac Tissue Regeneration

Heart attacks or myocardial infarctions are one of the primary causes of mortality and morbidity. Indeed, the primary cause of heart failure is left ventricular reconstruction following myocardial infarction.^191^ Dilation, hypertrophy, and tissue scarring are brought on by acute myocardial loss and the mechanical instability imposed by myocardial infarction and ischemia, particularly in the area of the left anterior descending the coronary artery.^192^ The capacity of self-healing injectable hydrogels to prevent pathological ventricular reconstruction and systematically preserve infarcted myocardium has already been demonstrated in rodent, ovine, and porcine models.^193^ Injectable hydrogels that heal themselves can be utilized in a variety of ways to facilitate cardiac tissue regeneration in addition to providing mechanical stability. While hydrogels equipped with growth factors, pro-angiogenic cytokines, miRNA, stem, or progenitor cells may trigger cardiac healing, hydrogels with antioxidative features can lower the oxidative stress in the ischemic myocardium.^194,195^ It has been proven that immunomodulatory hydrogels may lower the inflammatory response following a stroke.^196^ With injectable self-healing hydrogels, the infarct particular to each patient can be customized in terms of the amount, location, and rigidity of the implemented hydrogels. Quick and specific care for heart attacks is now possible because of the combination of noninvasive magnetic resonance imaging and finite element models, which have proven to be very helpful in witnessing the injured region and estimating the necessary tissue protection.^184^ A study focused on the hydrogel material used to treat myocardial infarction and concluded that cardiac pulsation, therapy strategy, and hydrogel creation were some of the crucial factors that play a role in the retention of injected hydrogels and treatment efficacy.^185^ A strategy for improving orientation control uses the pericardial cavity as an ideal hydrogel administration route.^197^ Initial clinical trials validated the efficacy of injectable hydrogels based on alginate to boost physical activity during myocardial infarction and avoid pathological left ventricular reconstruction. One study found that these hydrogels were well tolerated and did not cause any significant side effects; however, a different study found that the 30-day mortality rate was 8.6%. In another study, resilin-like polypeptides (RLPs), which contain biofunctional domains for cell–matrix interactions, were cross-linked to form RLP-PEG hybrid hydrogels.^198^ Cell viability assessments using confocal microscopy revealed that human aortic adventitial fibroblasts were successfully encapsulated in three-dimensional matrices and adopted a spread morphology after 7 days of culture.^198^ This necessitates the need for more research to create safe and effective therapeutic applications.

### 6.2 Spinal Cord Injuries and Ischemic Stroke

**Figure 5:**
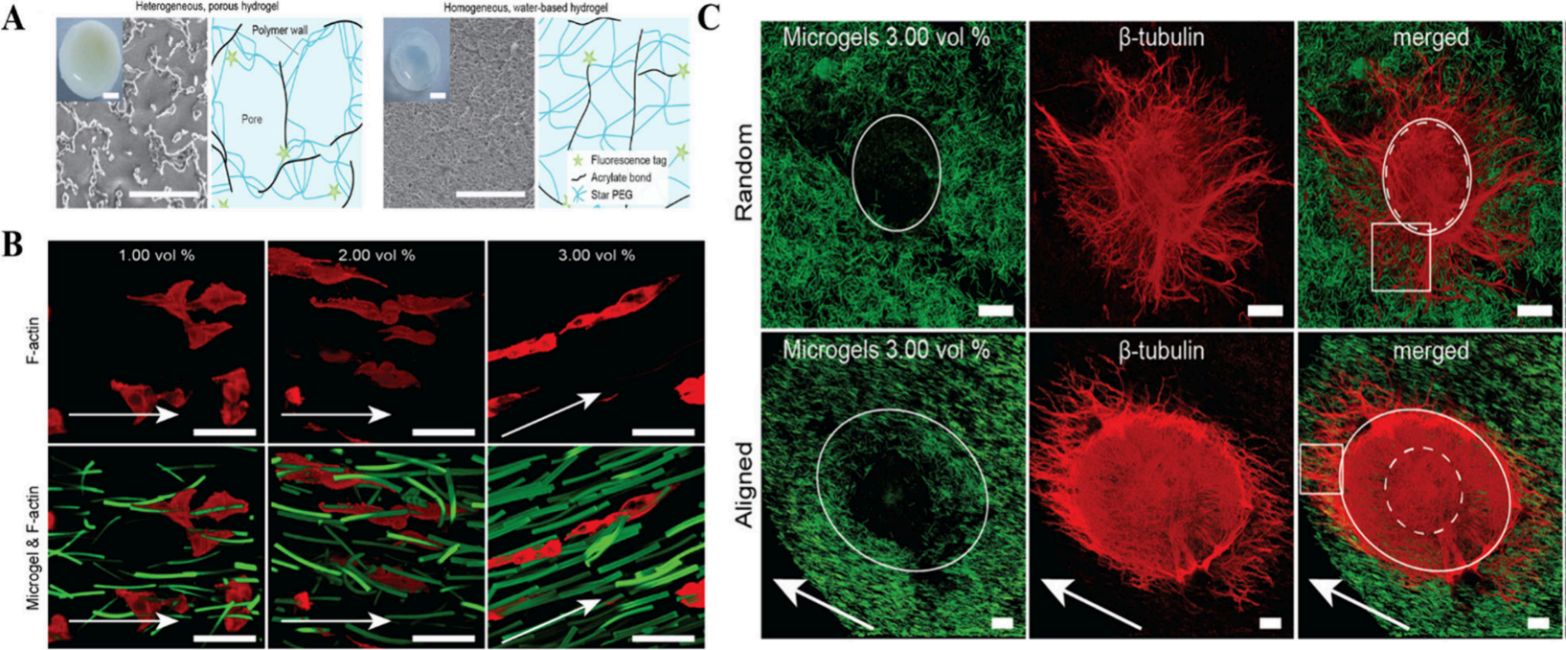
(A) Dilution of star-PEG-A (20 wt/vol %) with a nonreactive PEG-based diluent (here, 0.2 kDa PEG-OH) led to a visually white, heterogeneous hydrogel (left) consisting of water-filled micrometer-scale voids and nanoporous polymer regions, whereas by diluting with water, a homogeneous hydrogel (right) was formed with a transparent appearance and pores in the nanometer scale (scale bar is 2 mm in macroscopic hydrogel and 5 μm for FESEM). (B) Ability of the Anisogel to align fibroblasts when mixed with different concentrations (1.0, 2.0, and 3.0 vol %) of soft microgels, which aligned in a magnetic field of 130 mT. Premixed fibroblasts extended along the longitudinal microgel axis (green, fluorescein), visible by the stretched F-actin filaments (red, Alexa Fluor 594 phalloidin), depending on the microgel concentration (scale bar is 50 μm). (C) DRGs were positioned in hydrogels with 3 vol % microgels (green, fluorescein) containing random or magnetically aligned microgels. β-Tubulin staining (red, Alexa Fluor 633) revealed neurite outgrowth parallel to the aligned microgels (outside full white circle; scale bar is 200 μm). Adapted with permission from ref (201). Copyright 2017 American Chemical Society.

Some of the human body’s most delicate and clinically difficult-to-expose tissues are found in the central nervous system, and so far, only a few therapeutic alternatives are available. Injectable hydrogels with self-healing properties can address numerous challenges related to the medical application of traditional biological materials. As feasible, axons usually remain at the point of the damage in the injury of the spinal cord causing partial axonal rewiring. Connecting the wound to a supporting structure greatly aids in repair, but at this stage, invasive surgical procedures or implants may be difficult.^199^ Moreover, implants frequently lack sufficient adaptability to accommodate a diverse range of spinal cord damage. Injectable hydrogels with self-healing properties offer a novel approach to treating spinal cord damage in patients by facilitating less invasive delivery and filling. Self-healing injectable hydrogels have been successfully used to link spinal cord injuries and promote neovascularization and axonal development as studied in rat and porcine models.^37^ Injectable hydrogels can have geometric anisotropy developed in several ways by using magnetic orientation or the syringe’s flow field.^200^ In a study by Rose et al. on hydrogels containing magnetically oriented anisotropic microgels, the dorsal root ganglions showed guided development.^201^ Regardless of the microgels’ low volume concentration of less than 3%, where the geometrical constraint for orientation remains minimal, the resultant macroscopic unidirectional orientation is effectively detected by the cells, resulting in parallel nerve extension. This study gives up a new minimally invasive treatment option for spinal cord injuries (Figure 5). The creation of electroconductive hydrogels, which enable electrical signal transfer linking nerve cells and encourage their remodeling, presents an intriguing substitute.^202^ In addition to treating injuries to the spinal cord, researchers are looking at using self-healing injectable hydrogels to treat neurodegenerative illnesses including Parkinson’s and Huntington’s syndrome and to stop the degeneration of the intervertebral disc.^203^ Injectable hydrogels for the spinal cord should not enlarge in place because this could raise intraspinal strain and cause more damage.^36^ Ischemic stroke has relatively few treatment options available and is a leading cause of mortality and patient suffering. In addition to ischemic necrosis at the infarct site, a stroke can also release enzymes that break down the extracellular framework and ROS, which can result in secondary ischemia damage and tissue scarring that prevents repair.^204^ As they can be administered quickly and less invasively through the blood–brain barrier into the stroke space, self-healing injectable hydrogels are thought to be potential treatment options to reduce secondary ischemic damage. However, this is only possible if intracerebral fluid is extracted simultaneously to maintain a stable intracranial pressure.^205^ Injecting hydrogels filled with growth factors, neural progenitor cells, or erythropoietin right away into the stroke space have shown promising results in reducing subsequent ischemia damage and encouraging angiogenesis and neurogenesis following stroke.^206^

### 6.3 Bone Regeneration

Degenerative diseases, congenital issues, tumor excision, and trauma damage can all result in critical-size bone deficiencies that require medical intervention to heal. In vivo bone remodeling in medical settings is still commonly achieved through the use of autografts, allografts, or bioceramics, which can result in donor site infection and suffering.^207^ These limitations highlight the need for innovative bone graft materials that can be utilized to fill patient-specific deficiencies with less invasive procedures, encourage bone repair even for broader deficiencies, and avoid eliminating nonessential surgery.^208^ Numerous studies on self-healing injectable hydrogels that fix bone damage and promote recovery have been reported recently.^209^ These hydrogels are frequently used in conjunction with medicines, growth factors, nucleic acids, ions, and stem cells to enhance bone recovery.^210^ The poor mineralization capacity of hydrogels or their failure to precipitate the ions of phosphate and calcium into hydroxyapatite crystals poses a challenge to the regeneration of bones. The primary way of encouraging bone mineralization is the addition of hydroxyapatite or calcium phosphate particles.^210^ In addition to their pro-angiogenic behavior and ability to rebuild bone via apatite generation and ion release, bioactive glasses are also frequently used in hydrogels.^211^ Additional strategies consist of matrix vesicles, soaking hydrogels in calcium phosphate solutions, and adding enzymes that promote the accumulation of bone mineral.^32^ In particular, the newly identified impact of hydrogel viscoelasticity on the maturation and activity of cells holds immense potential for regenerating bone.

## 7 Vitreous Substitute and Ocular Delivery

**Figure 6:**
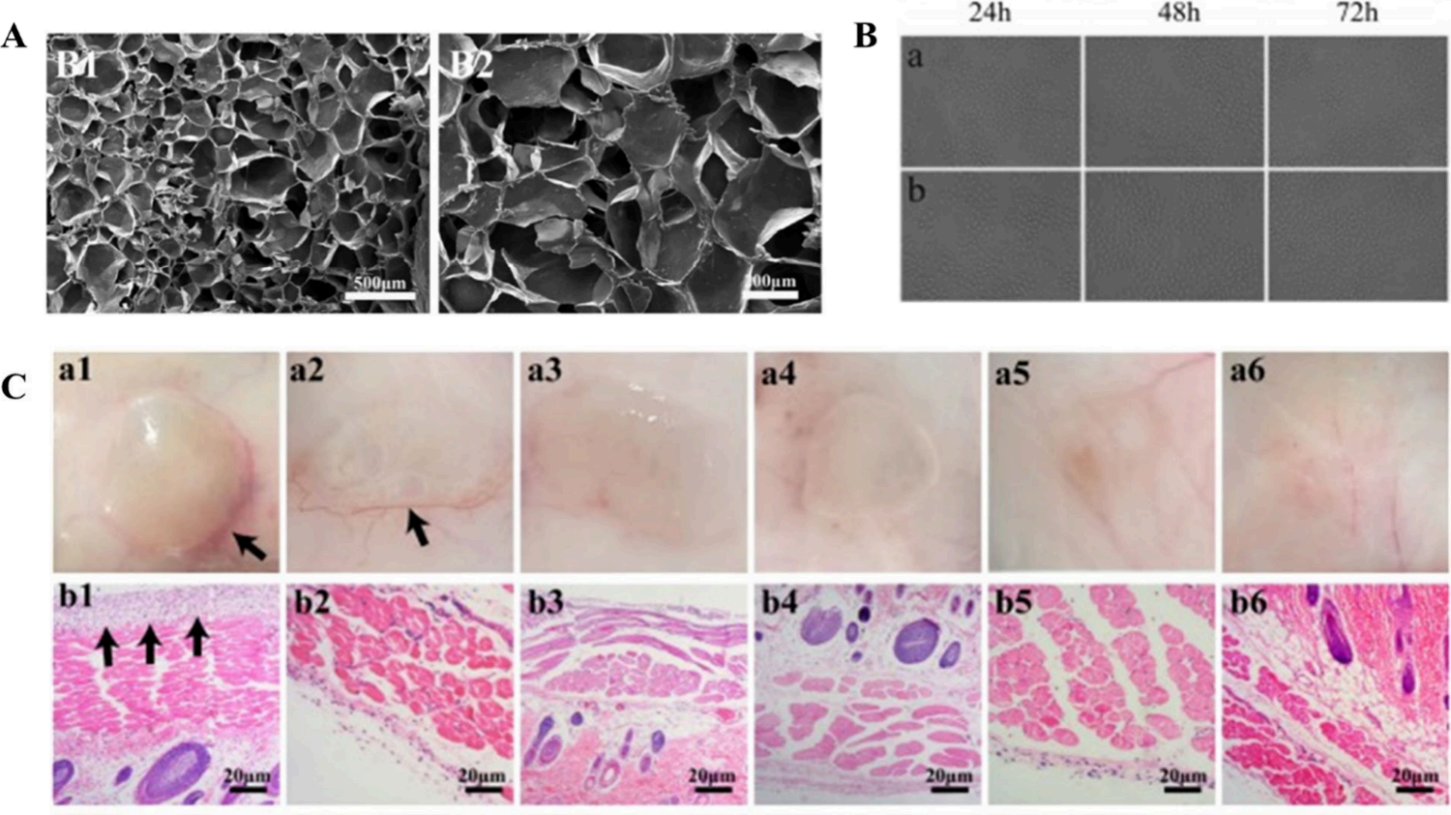
(A) Scanning electron microscopy image of chitosan and hyaluronic acid hydrogel at 100× and 200× magnification. (B) Biocompatibility of the hydrogel as observed from a L929 cell growth study in (a) the control group and (b) hydrogel treated. (C) In vivo biocompatibility of hydrogel on skin tissue at week 1, 2, 3, 4, 6, and 8 (a1, a2, a3, a4, a5, and a6, respectively) after hydrogel treatment. H&E stained images of subcutaneous tissue at the injection sites at the same time duration (b1–b6). Adapted with permission from ref (214). Copyright 2020 Elsevier Ltd.

A frequently performed ophthalmological surgery used in the cure of retinal detachment, macula, or diabetic retinopathy as well as for the elimination of vitreous hemorrhages and floaters is vitrectomy, which involves the removal of part or all of the vitreous fluid. Syringes are being used to carry out vitrectomies in a less intrusive way.^212^ A gas or saline is typically used as the replacement for the lost vitreous fluid to sustain intraocular pressure.^213^ Injectable hydrogels made of chitosan–hyaluronic acid that can repair themselves have surfaced as viable long-term vitreous replacements that increasingly mimic the physical and diffusive characteristics of vitreous humor (Figure 6).^214^ In addition to having sufficient mechanical qualities, injectable hydrogels for vitreous replacement should have refractive index amounts and long-term visibility comparable to those of the vitreous humor. Large-scale vitreous fluid vitrectomy can disrupt the ocular oxygen equilibrium, leading to oxidative damage and the development of cataracts. Hydrogels enriched with antioxidants may inhibit the growth of such cataracts after vitrectomy.^215^ Although eye drops are a simple topical ocular means of administration, their quick disintegration and physiological obstacles frequently prevent them from properly delivering dosages to the internal eye.^216^ Injectable hydrogels that repair themselves have been used to provide antivascular endothelial growth factors and administer chimeric antigen receptor cells intraocularly to cure retinoblastoma or inhibit neovascularization.^217^ One of the main sources of degeneration of the macular tissues is ocular neovascularization, which may occur after an injury or infection. Self-healing injectable hydrogels can deliver a reliable, long-lasting medication dosage that is necessary to stop degenerative eye disorders.^40^ Medicines are contained in biodegradable microspheres scattered throughout the hydrogel matrix to extend medicine distribution durations to as long as 6 months. Additional ways for minimizing macular degeneration include the introduction of stem or progenitor cells or the administration of antioxidative hydrogels.^218^ Additionally, favorable results were found using cell packing therapy, which involves administering hydrogels containing genetically modified cells that frequently express antivascular endothelial growth factors to prevent ocular neovascularization over the long term.^219^

## 8 Summary and Future Scope

This review reveals the wide range of applications of hydrogels, including wound healing, antibacterial activity, delivery of drugs for cancer therapy, tissue engineering, and regenerative medicine. In addition to the fundamental technological advancements in hydrogel research, more fields have the potential to use hydrogels. While some hydrogels, like synthetic hydrogels (poly(vinyl alcohol), polyacrylamide, polyethylene glycol hydrogels, etc.), do not have good biodegradability and biocompatibility, these properties can be changed by changing the functional group of the hydrogel or by adding natural polymers. Additionally, several protein-based (keratin, collagen, gelatin, casein, and resilin) and peptide-based (including π–π stacking, β-sheets, and α-helices) hydrogels have been developed and used in biomedical applications.

In 3D cell culturing and tissue engineering, semisynthetic hydrogels can resemble the extracellular matrix of biological tissue while optimizing the drawbacks of both natural and synthetic hydrogels. The clinical utility of photoresponsive hydrogels should be assessed regardless of their superior drug delivery capabilities under stimulated light radiation. The functional hydrogels’ bioactive characteristics aid in the healing of wounds. Antibiotic- and metal-nanoparticle-loaded hydrogels have effective antibacterial qualities that assist in treating infections and combat biofilms.

In the past decade, scientists have created a wide range of smart biomaterials that can recognize, respond, and adjust to changes in their surroundings. The creation of these “smart hydrogels”, also referred to as “stimuli-responsive hydrogels”, as functional materials has been a remarkable advancement in the field of biomaterials. These smart hydrogels can change both structurally and volumetrically in response to external stimuli, which opens up a wide range of possibilities for multifaceted uses in technology. Typically, absorbent to superabsorbent materials that respond to even the smallest changes in pH, temperature, electric field, ionic strength, chemical species, and biological conditions can be used for smart hydrogels development. Since the size and structure of biological tissues affect their function, one significant challenge is replicating tiny characteristics in physiologically relevant sizes. For example, when constructing thin hollow tubes such as blood arteries, kidney tubules, and nerves, several ways have been suggested to overcome the dimension constraints in scaffold preparation. The biocompatibility of synthetic polymer hydrogels with encapsulated cells and the host organism presents another difficulty. An attempt is being made to make polymer materials more biocompatible.

Hydrogels generated from polysaccharides are applied as biomimetic wound dressings, stimulating macrophages to induce nonspecific immune system activation. Advanced polysaccharide hydrogels require innovation to achieve physicochemical properties that may be customized, making them effective drug carriers. Using polysaccharides, which may be produced in an efficient and environmentally friendly manner, it is possible to create perfect hydrogels with strong healing properties and promising performance. More investigation is necessary to give more exact control over the gels’ physicochemical characteristics, which have a strong biomodulatory effect on wounds. Further developments are necessary to achieve the successful application of hydrogels with cell attachment in the wound healing process. Limited hydrogels are available commercially for use in tissue engineering, drug delivery, and 3D scaffolds for clinical purposes. However, hydrogels will find greater use in clinical practice as a result of our growing understanding of the mechanical strength, elasticity, and biological makeup of the extracellular matrix.
